# Legal aspects of service robotics

**DOI:** 10.1007/s10202-012-0115-4

**Published:** 2012-11-27

**Authors:** Thomas Dreier, Indra Spiecker genannt Döhmann

**Affiliations:** Center for Applied Legal Studies (Zentrum für Angewandte Rechtswissenschaft, ZAR), Karlsruhe Institute of Technology (KIT), Vincenz-Prießnitz-Str. 3, 76131 Karlsruhe, Germany

**Keywords:** Winner, Hermann, Hakuli, Stephan, Wolf, Gabriele

## Abstract

The emergent use of service robots in more and more areas of social life raises a number of legal issues which have to be addressed in order to apply and adapt the existing legal framework to this new technology. The article provides an overview of law as a means to regulate and govern technology and discusses fundamental issues of the relationship between law and technology. It then goes on to address a number of relevant problems in the field of service robotics. In particular, these issues include the organization of administrative control and the legal liability regime which applies to service robots. Also, the issue of autonomy of service robots is discussed, which cannot easily be answered under the existing, human-centered legal regime.

## Abstract (de)

Der zunehmende Einsatz von Service-Robotern in immer mehr Bereichen des gesellschaftlichen Lebens wirft eine Reihe rechtlicher Fragen auf, die es bei der Anwendung des bestehenden Rechtsrahmens auf diese neue Technologie zu beantworten gilt. Der Artikel gibt einen Überblick über das Recht als ein Instrument zur Technikregulierung und erörtert grundsätzliche Probleme des Verhältnisses von Technik und Recht. Nachfolgend wird eine Reihe rechtlicher Fragestellungen auf dem Gebiet der Serviceroboter angesprochen. Dazu zählen insbesondere die Organisation und Ausübung einer verwaltungsrechtlichen Kontrolle wie auch Fragen der Haftung von bzw. für den Einsatz von Service-Robotern. Schließlich wird auch das Problem des rechtlichen Umgangs mit der Autonomie von Service-Robotern angesprochen, das sich im geltenden, auf den Menschen ausgerichteten Recht nicht ohne weiteres lösen lässt.

## Challenges

Robotics as an evolving technology combining efforts from computer science, mathematics, electrical engineering, mechanical engineering, and other technical disciplines has developed for a considerable amount of time. The social sciences have, however, been little involved in the process of developing and implementing the new technology. An assessment by the social sciences, by law, by economics is long overdue. This is particularly true for the differentiations in this field: Robotics as such might be considered; the field of service robotics as a particular area is still less considered with its particularities. This is also very much true for the legal assessment of the challenges to society; research and scientific reflection are just beginning. An additional problem is caused by the necessity of legal analysis having to be based on the relevant law and thus necessarily having to restrain itself to a national or at best European analysis unless it refrains from concrete evaluations.

However, we find that many of the legal problems caused by the emergent use of service robots in more and more areas of social life can be addressed without having to refer to concrete norms and particular court rulings, considering the current state of legal research in this field. Therefore, the following text will give an overview over some of the urgent questions that arise for the legal field by the far-reaching use of service robotics. Sometimes, references to particular states of legal thinking are made based on German and/or European standards. However, most of the questions addressed are not bound to a background in this legal environment. The paper will therefore start with an overview over law as a means to regulate and govern technology including general problems deriving from this area (Sect. 1) and then address a number of relevant problems in the field of service robotics (Sect. 2). We follow a systemized, life cycle approach that looks at legal questions beginning with the design of robots and closing with the deliberate end of a robot, its dedication to become waste. It is our intention to raise the relevant questions; sometimes, we will offer first-step analysis to address the identified problems, but we refrain at this stage from developing explicit and elaborate solutions.

### Introduction: Law and technology

#### The many functions of law

Law fulfills a number of functions. Some of these functions gain particular relevance when it comes to assessing the technological impact of employing service robots.

First and foremost, the protective function of law plays a major role. Ever since the technology debates in the 1960s, this particular consideration has gained substantial influence on the state regulation of private issues: The state’s obligation to protect has continually been emphasized and increased. By means of this legal responsibility, the state is forced to create and maintain societal framework conditions beyond the general state–citizen relationship. This shows that an obligation exists to safeguard at least a minimum of protection.

When continuing along this line of thought, the state also acts as guarantor of an individual freedom above and beyond the state–citizen relationship: It defines and protects spheres of freedom for the individual from intrusion by private third parties. State regulation itself is a prerequisite for enabling an individual to exercise his civil rights and liberties. In turn, the guarantees of freedom and protection themselves are interconnected. This becomes particularly clear when considering the guarantees motivated by the Social State Principle rooted in Art. 20 I GG, the German Constitution, the Basic Law, of the national constitution. When the state’s duty to protect is understood in such a way, it requires the state to facilitate opportunities for human actions while minimizing the risks for others that are caused by these actions.

Both aspects of this obligation to protect are highly relevant when considering the use of service robotics: The employment of robots in the field of nursing may help the elderly to protect and maintain their individuality by enabling them to remain in the surroundings which they are accustomed to, with all the familiarity and privacy this implies. Also, the use of logistics facilities may keep individuals from having to perform tasks which put their health at risk. Simultaneously, however, it is important to implement legal regulation protecting the beneficiary of robot services, as well as third parties, from being imperiled by the use of such technology. Possible dangers include the robots destroying things, compromising privacy or being perceived as threatening. The question of to what extent the state must act for reasons of public welfare also belongs in this category.

Another function of the law that gains relevance in connection with service robots concerns construction and planning security. Service robots are highly complex investments. Their successful implementation requires that certain conditions are present, such as a living environment suitable for robots or level streets expedient to robot locomotion. These conditions can be induced by means of legal regulation. This enables technology to reach a level of functionality so as to lead to good, possibly even optimal, and socially compatible use.

Finally, the law increasingly ensures the functioning of markets within the economic system. In this area, governmental action is no longer legitimized by defense against threats but for the reasons of market failure. This is also connected with the state’s function as guarantor of freedom: A malfunctioning market cannot create a balanced allocation of goods, does not spawn innovation, produces negative external effects, and leads to a decrease in quality. In consequence, the exercise of freedom is hampered seriously.

The use and spread of service robotics makes demands of the state in all of these functions. The protective function necessitates the defense against the potential hazards of extensive use: The employment of service robots in road traffic, for instance, can endanger individuals and the general public not only in cases of malfunctioning, but in the course of normal operation as well. At the same time, individual freedom is enhanced by the possibilities that the use of service robots offers: In times of limited government solvency and efficacy, especially concerning the health care and pension systems, and an aging population, a shift from non-functional personal services to functional machine-controlled services might even give the protection of individual freedom and safeguard of dignity a whole new meaning. The functioning of the market for service robotics, should it develop similarly to industrial robotics, would be jeopardized without government intervention: A trend toward monopoly caused by network effects and significant asymmetries of information lurks; negative external effects are not internalized.

Aside from its regulatory function, legal regulation is able to control human behavior. This power can be exercised by regulatory means, in other words through commandments or prohibitions. Other instruments such as information or incentives may also be utilized. Most notably, however, there are norms dealing with the arrangement and distribution of risk between private parties.

Potential addressees of regulation range from individuals employing service robots, to manufacturers and developers of the technology, to industry associations, trade unions as well as state institutions such as health insurance.

#### Law and technology: methodical issues

Methodically, behavioral control through law encounters a whole number of problems. These shall be outlined briefly within the scope of this article:

- To begin with, laws are pure “ought-norms” (Sollensnormen), which means they describe a certain conduct that the addresses are to comply with. Even the determination of sanctions in case the legal norm is not adhered to, is in and of itself a legal norm and therefore also no more than an “ought-norm.”
- In order to be effective, law additionally requires practical enforcement. This leads to questions concerning means of enforcement and enforcement readiness as well as questions of acceptance on the part of the addressees.
- Legal norms are not the only, probably not even the most effective means of influencing human behavior.
- Being a societal subsystem, law does not exist in isolation. Much rather it reflects the predominant fundamental views of society and its concepts of justice. It is therefore not able to govern circumstances relevant to society without being influenced by the circumstances themselves. There is a certain interdependence which imposes limits on legal regulation.
- Such interdependence is also characteristic of the relationship between technological progress and its legal regulation. Only within limits is law able to influence the development of technology, it might even constrain its usage. Hence, it is technology that sets the tone for law. In essence, law—with the exception of an all-out prohibition—structurally tends to react to technological developments rather than steer them proactively.
- Because the evolution of technology, the range of its possible application and the consequences of its employment are difficult to predict, creating legal regulation means making decisions in the face of uncertainty. This hampers the effectiveness of legislation and can be detrimental to important preconditions (acceptance; legal certainty; …). At the same time, during periods of uncertainty, law can become a tool that primarily protects the status quo when new developments are forced to prove their superiority over contemporary standards (e.g., in liability law).
- On the other hand, the symbolic nature of regulation should not be neglected: The mere existence of a rule has its effects even if the likelihood of its enforcement is low.
- The problem of linguistic and pictorial metaphors is also connected to lawmaking and the application of norms.

Nonetheless, the following is not to be neglected:

- Legal norms are a major—though admittedly not the only—instrument utilized by policy makers in attaining their objectives.
- With contemporary law, society has created an apparatus which, at least in its essentials, has been approved of over the years and whose fundamental structure has proven itself sufficiently stable and adequately flexible.
- Law enables predictability through legal certainty and through its binding quality. In the extreme, this can lead to law constructing reality.
- Law can promote the acceptance of technological innovations.
- Positive law requires a political decision by the bodies designated for the process and thereby gains binding quality for (private) addressees. However, not making law also has its effects, since it ultimately leaves the power of decision making to the (private) decision makers, Art. 2 I GG.
- Law creates a compromise between various individual interests and between general welfare concerns and individual interests.

#### Law and service robotics: the approach

In light of this—and not simply because of the validity of existing legislation—the first step involves applying the existing norms to the novel technology of service robotics and then assessing the consequences with regard to their appropriateness (“current state analysis”). Only when—and as far as—the consequences prove to be inadequate and/or regulatory deficits exist, does it become necessary to take a second step and adjust the law in light of the factual as well as the predicted technological development. Such a need for amendments may go beyond the adjustment of individual norms and—considering the automation and autonomization of service robots—could well have an impact on basic legal concepts. Possible issues include the question of a uniform “robot law” or sector-specific provisions (“target state analysis”).

### Issues/questions that are open to and in need of regulation

#### The fundamentals

Hitherto, existing literature has primarily dealt only with the aspect of liability for robots. On occasion, a robot’s legal capacity and its capacity to act are addressed. Usually, value- and scenario-based discussions face off. Similarly, the conflict between individual and general interests crops up time and again.

With that said, the normal operation of robots (capacity to act) as well as detrimental unintentional or knowingly accepted consequences of their employment (accidents) has been considered. The manufacture and sale, however, remain undiscussed. Much the same is true for the termination of operation and disposal. If one considers service robots comprehensively, beginning with their design/manufacture and their sale up to their employment and disposal, in other words if one considers service robots systematically, focusing on their life cycle, a whole number of legal issues arise, going beyond those of autonomy and damage liability. These issues shall be addressed in detail below.

One key issue concerns the compulsory use of service robots or rather the governmental incentivization aiming to promoting the employment of such robots: To what extent can the state make the use of robots mandatory? Depending on the scenario, this obligation might be based on various legal reasoning (principle of dignity, paternalism, effectiveness, intrusive nature of incentivization, traits of robot operators/users…). In areas where state intervention is prevalent anyhow—such as in health care, for example—this is more likely to occur than in areas with low governmental control, which are largely subject to private decision making. For most of these areas, the question is one of determining to what extent the statutorily envisioned employment of robots is compulsory or whether the issue is open to inter-party negotiations, meaning that the legal norm can/is only to be viewed as a “default rule.”

Finally, another way to connect law and technology could be to implement some of the binding rules that are not subject to private autonomy by prescribing compulsory elements of construction and making them part of a service robot’s makeup at the outset. In this way, compliance and enforcement deficiencies would be prevented from ever developing (“law in/ex machina,” “law through technology”).

#### General rules and concrete problems

Determining which legal norms are applicable to an individual case and what the legal consequences of the assessment are requires knowledge and consideration of the intricacies present in individual cases. Since these can not be delineated completely beforehand, the following deliberations will focus on so-called scenarios, that is to say on standardized and typical applications. Scenarios exemplify. Yet, at the same time, they restrict. For any peculiarity which the scenario does not take into account will not be covered by the legal reasoning. In order to address such problems as might arise from the use of service robots in situations not covered by the specified scenarios, the entailing legal problems shall at least be pointed out.

#### Regulation: paternalism or market?

The previous considerations have not touched upon the question whether or, more importantly, why service robots should/must exist. When addressing this issue, the question inevitably arises whether or not state regulation promoting the use of robots and preventing potential negative repercussions should/must exist in the first place.

Ultimately, however, law is confined to implementing political decisions, which in turn are, or at least should be, based on ethical considerations. Therefore, public policy is often times not the manifestation of some superior set of values but rather of a continuously self-affirming and dynamic evolution of values. One central theme is the aspect of general welfare which, according to legal understanding, seems not to be part of the accumulation of individual interests, as becomes apparent when the sustainability of decisions is taken into account.

Another question is to what extent the government, even when making a positive basic decision, should be allowed to introduce a rule that is binding for everyone alike (total paternalism), or whether it should rather accord an individual the liberty of choosing the objective or prioritizing an objective, or at the very least grant the opportunity to “opt out”. Speaking in terms of ethics, the question raised is the following: Who may determine the objectives? If necessary, one must differentiate between primary and secondary objectives. In respect to robotics, one issue concerns the extent to which objectives should be set and selective choices may/should be made by the constructor.

#### Governmental decision making in the face of uncertainty

A central element of the legal supervision of technology lies in the necessity of introducing regulation without having a secure and reliable grasp of the present and the future. The development of technology in particular, as well as its consequences and its ties to other developments frequently eludes prediction with any degree of certainty. In light of the partially substantial infringement of fundamental rights that legal regulation entails, this leads to problems of justification, particularly as far as the emphasis to be put on unreliable knowledge is concerned: Is it to be factored into the decision in positive or negative reading? What mechanisms can aid the non-knowledge decisions, which of these can not be used in cases of uncertainty? To what extent is the precautionary principle applicable? Do special supportive mechanisms exist that could respond to the progression of knowledge? And above all: May the state impose restrictions under conditions of uncertainty when and if inaction is just as likely to lead to the encroachment of fundamental rights as taking action is?

#### Regulatory instruments

##### Focal points of the past discussion

If one acknowledges a minimum of governmental control in handling service robots, the question arises, which regulatory instrument is best suited for achieving the individual regulatory goals. By focusing on autonomous robots’ legal capacity to act and on the liability for accidents caused in the course of their operation, the fact has been neglected that the body of law is not confined to norms concerned with determining legal capacity and to supplying rules on liability as the minimum requirement for orderly social interaction. Moreover, it offers a variety of additional provisions performing a whole string of different functions; especially from the point of view of technology law, a whole number of other aspects concerning the regulation of service robot employment emerges.

##### Instruments of technology law

For the use of service robots, one can draw upon the entire apparatus of legal regulation that technology law has in store and which was originally developed for other, already existing technologies.

This includes—with no claim to being exhaustive—for private law:

- Traditional indirect control through liability rules
- Imputation rules, and the implementation of liability for fault or strict liability
- The establishment of duties of care
- Compulsory insurance systems
- Direct influence through the establishment of standards and the control of general terms and conditions.

Civil law sanctions with the purpose of deterrence exist only in rudimentary form at the present time.

As far as public law is concerned, the available arsenal is significantly more extensive. It includes:

- Instruments of precautionary supervision (facility registration and authorization; proofs of conformity)
- Instruments for the surveillance of companies
- Instruments of knowledge acquisition, knowledge support, and knowledge dissemination for dealing with uncertainty; monitoring of knowledge development
- Obligations to upgrade and take measures of precaution
- Standardization guidelines
- Liability and imputation rules as preventive instruments of control
- Other instruments of incentivization
- Learning: information/compulsory labelling/warning notices/test obligations
- Institutional requirements such as ombudsman/appointee/federal agency/routine standard examination
- Obligations concerning facilities and operators, behavioral obligations

##### Governance and control

This list shifts the instrumental view from traditional commandments and prohibitions—and thereby from the mere application of existing liability regimes—to the entire field of governance and control, including a look at the actions or reactions of the parties involved. Possible options include, but are not limited to

- a mixed control made up of admission/control and liability sanctions (if necessary, by means of criminal law)
- a direct control, that is, the formulation of binding regulation primarily by public (regulatory) law
- an indirect control through compelling or negotiable norms of private law
- the involvement of private citizens in the regulation process by way of regulated or unregulated self-regulation (for example through private rule-making; the creation of certifications)
- the inclusion of non-governmental organizations (NGOs) in the decision-making process
- the consideration of new forms of civic participation, transparency, and other procedural measures.

## Specific problems of employing robots in various settings

### Terminology

To start off with, a clarification of the terminology seems called for: When speaking of service robots, the term “service” does not correspond to the word as it is used in legal discourse. Accordingly, German civil law differentiates between “Dienst” and “Werk” (§§ 611, 631 BGB) depending on whether the commitment is construed to involve simply the taking of action (i.e., trying to achieve a certain result) or having the duty of actually achieving specified results. The achievement may be the result of a manufacturing occupation (tailoring a suit) or of a service (cutting hair). In telecommunications law, the term “Dienst” describes the rendering of telecommunication services (at least—not quite recursion-free—this can be inferred from the definition of “Diensteanbieter” in § 3 Nr. 6 TKG). In European law, one distinguishes between the “free movement of services” (Art. 56 et seq. TFEU) as a protection of the free movement of services and the “free movement of goods” (Art. 30 et seq. TFEU). It is subsidiary to the latter and also to the “freedom of establishment” (Art. 49 et seq. TFEU).

### Problematic issues in the systemic life cycle

In general, service robots start their existence on the drawing board: They are developed beyond pure research based on presumed market demand. As early as this stage, the development of service robots is accompanied by legal regulation such as occupational safety provisions or norms of substance law. Further legal questions come up from the time of their concrete employment right up to their disposal. The subsequent deliberations therefore follow the life cycle of a service robot and elaborate upon the typically occurring problems at their respective stages in order to present a thoroughly comprehensive picture.

#### Planning, manufacture, and sale

Service robotics is considered to be a highly innovative field of technology. Innovation, however, is prone to governance effects, for example, by state programs, but also by law. Its further development is therefore also dependent on the environmental factors investing into innovation in this area. In how far such innovation incentives are generally useful, is at least questionable. A more concentrated and more precise, specifically problem-oriented approach might be one of the effects of an interdisciplinary technology assessment process. Such an approach would—among other aspects—address the particular needs of a multiplicity of users.

Legal rules do not only create incentives for innovation processes in service robotics as such, but also consider legal problems on a prior or more general level, for example, they lower market entry barriers or counteract existing competitive disadvantages.

In line with the freedom of research, a number of facilitations and privileges having to do with planning and construction are available to the original “inventors” of service robots. These range from a special protection of their inventions by means of employee invention rights and copyright, to the granting of patent and trademark protection. However, these privileges end when a service robot ceases to be an object of pure research and is to be put on the market, instead. When a robot is put into circulation, the notion of protecting the researcher is superseded by concern for the safety of the general public.

Thus, a number of different restrictions on the manufacture and sale of service robots are conceivable. These cover the design process as such, the specific manufacturing process, as well as the sale in its function as the means of putting the robot into circulation. Such restrictions may stem from specific statutory rules, for example, the applicability of the Medial Devices Act to the employment of nursing robots or may arise from quite general regulations as well. These general issues may include questions concerning a legal basis for introducing an authorization requirement, a general power of intervention of the authorities, fault-based and strict liability up to construction, and product monitoring obligations which must already be observed at the stage of planning and manufacture. Considerations concerning innovation processes belong here as well: How best to stimulate lead innovations in research and how best to accommodate the different and independent desiderata of research and normativity? Last but not least, a thought must be given to the importance, development, impact, and design of standards that enable an extensive and therefore profitable application in the first place. Such an examination is not promising without a continuous reassessment of the standards: Whose standards are to be applied? An engineer’s standard or rather a user’s standard?

Considering these fundamentals, specific questions concerning the development and sale of service robots are pressing. To what extent must the development of service robots comply with privacy standards in the phase of construction? To what extent must the final control remain with the user, particularly with the employment of service robots in various surroundings?

#### Regulation of normal operation

Many of the questions in the field of development, manufacture, and marketing refer directly to the regulation of service robots’ normal operations. This area requires the development of conditions of use and of framework conditions. The conditions of use are connected with issues such as: Who is responsible for the commissioning, which steps in learning and adjustment are to be required? How best to deal with the special characteristics of operators (children, the elderly, the sick…)—by imposing general and specific eligibility requirements for the use of service robots? Are there special procedural requirements that must be heeded, such as warning labels or participatory elements in determining fields of service in order to safeguard the rights of users or third parties including their dignity and individuality? Do these legal positions require informed consent prior to employment? How can shared, multiple or parallel use be accommodated by legislation, particularly when the distinct characteristics, dispositions and interests of multiple users need to be taken into account?

Framework conditions concern the configuration of the environment in which service robots are employed, for example, infrastructural tasks such as the elaboration and restructuring of construction law, for instance, or specific conditions for the employment of service robots. Even provisions in road traffic regulation are not out of the question if service robots are to be classified as motor vehicles. Furthermore, fields such as privacy law and personality rights will need to be revised if it becomes necessary to gather, store, and utilize personal data of the user or third parties, especially if this is carried out with the use of cameras for the purposes of localization. Since service robots are often used in systems, this can quickly lead to a de-facto obligation to feed data into systems, which allows third parties to access highly personal information along with the risk of it being analyzed and exploited. Company-related data can be endangered as well when participation in a system—for example, in the field of advanced driver assistance—entails the disclosure of information.

The key issue of attributing the use of service robots must be answered by the law: Who is their employment legally to be attributed to, who is legally to be viewed as the provider of services performed by the service robot: Is it the person whom the service is provided for? The person who put it into operation? Or possibly a third party who encouraged its use and/or paid for its services or made it available, such as the health insurance provider? Or should one possibly consider if the robot has not its own legal personality? Does legal liability ensue from the operation of equipment or from personal fault? The more service robots are incorporated in a system intimately, and thus the more people have access and use, the harder it gets to make an association and the harder it becomes to make an assignment to legal categories.

The particulars of a specific service robot’s employment are crucial for a regulation of normal operations. What differences appear when it is used in the private as opposed to the public sector? Should this differentiation perhaps be expanded and intensified, for instance in respect to privacy and absolute privacy, or probability and typicality concerning the use of service robots?

Legally, all of these issues lead to the formulation of permit and authorization requirements, both in respect to the type of service robot and in respect to its specific, individual employment. When a technology is subject to this kind of basic regulation, it inevitably entails duties of care and surveillance during operation, for example, the monitoring of operational safety or maintenance.

#### Accidents/malfunctions/unintended consequences: duties of care and standards of liability

Providing services in the designated manner constitutes the normal operation of a service robot. However, malfunctions and damages are to be expected. It is also to be expected that the use of service robots will lead to negative aftereffects, unpredicted breakdowns, and unintended side effects of some sort. These consequences of employing service robots also need to be taken into account by the law. They have been the focus of legal considerations of using robots in general.

When dealing with such unwanted effects, one must consider first and foremost that not only is it the user of a service robot that comes into contact with it/its sphere of activity, but the same is true for third parties, such as guests or traffic participants. Legal regulation should be open to modification in many scenarios of service robot employment because the potential damages are comparably low, at least when compared to the use of genetically manipulated plants or nanoparticles. While many developments may not be predictable and all players act in considerable uncertainty, the possible damages and the potentially aggrieved parties are mostly foreseeable. The susceptibility to damages is limited anyhow as far as original damage is concerned, such as the consequences of a non-functioning service robot. In contrast, damages affecting the surroundings depend on the concrete circumstances, such as the degree of damage done to someone else’s property caused by misguiding the robot. The potential for catastrophe rises with the ubiquitous use of service robots; the vagueness with which probabilities can be assessed follows the same pattern. This holds true especially when systemic effects are to be feared, for example when an extensive and intensive interaction of various service robots is envisaged, as is the case in advanced driver assistance systems. A single error in a single component of the system could potentially lead to extensive, even catastrophic damage. Therefore, compensation systems that differentiate based on damage potential and systemic involvement need to be developed. At least in cases of physically bound employment, the number of claims that will have to be dealt with should be limited.

All pertinent provisions build upon an existing, sophisticated liability regime, though its applicability to service robots with their marked technical independence will need to undergo thorough examination. Particularly, product liability law which is based on European legislation could offer some valuable insights. However, the statute seems not to be applicable to the provision of services.

Liability rules induce manufacturers and users to take special care—at least when those rules involve so-called fault-based liability, meaning that liability ensues only as a result of negligent or deliberate action. The contemporary state of liability law reflects this development: The events giving rise to a claim for damages are preponed in so far as the breaching of a duty of care prior to the actual occurrence of a damage-inflicting incident may already suffice for establishing damage liability. In this respect, the particular actual conditions of service robot employment need to be taken into consideration when establishing a duty of care; especially, obligations to inform have received a prominent role. However, much of the information can not even be processed in the short time available. On the one hand, comprehensibility is a significant requirement when it comes to the operation of service robots. On the other hand, it may lead to a drop-off in the controllability—and consequently in the adaptability—of service robots. Moreover, vagueness in communication with the robot must be handled. Thus, liability rules can promote standardization and technical formalization. Simultaneously, however, they can diminish the impact of service robots. This also shows that the users of service robots can likewise be encumbered with duties of care, the non-observation of which could lead to a partitioning of liability.

When a service robot causes harm to one of the contracting parties in the course of a contractual relationship, liability can also result from contractual obligations. These contractual stipulations may differ substantially from the statutory liability regime. Inter alia, possibilities for waiving liability are quite conceivable, such as indemnifying the developer or provider of service robots.

On the level of public regulation, liability norms are a significant and historically proven mechanism, particularly in technology law. Since disaster prevention can scarcely be left to individuals, especially in relation to systemic effects, this particular task is one that the legislature has to deal with. It must strike a balance between public welfare and the interests of individuals. This includes establishing a healthy balance between an individual’s interest in not coming to harm and the innovative interest in generally facilitating developments or the interest in a functioning economy or health care system in times of an aging population, high unemployment rates, and an increased number of people in need of care, particularly in cases of dementia. Also, an individual limit of sacrifice can be set. Frequently, this is accomplished by statutorily declaring potentially risky behavior as being principally admissible—such as is the case for placing genetically modified organisms onto the market, but coupling this freedom of action with increased liability sanctions such as strict liability: liability is incurred purely for engaging in potentially dangerous behavior, regardless of personal fault.

When designing a liability regime of public nature, the legislature has to take a number of basic differentiations into account that have now been touched upon repeatedly and which effect not only the regulation of normal operations but the area of liability as well. So far, the law has barely specified the employment of service robots in the private versus the public sector, the influence of the user and the victim upon the circumstances of a service robot’s employment or the particular characteristics of users [such as age, comprehension (particularly in cases of dementia)]. Thus, minors are accorded special protection and a guardianship law is in place that can apprehend developments in old age; beyond that, however, specifications are absent.

Finally, systemic effects can lead to the nonuse of service robots becoming a subject of discussion. This applies to health law, for example: If the employment of service robots disburdens public health care, then to what extent is an individual’s autonomy concerning their (non-) use to be respected?

When a private or public liability regime is in place, governing impact on developers, distributors, and users of service robots ensues: Countermeasures are taken to ward off liability and preventive steps are taken to avert the occurrence of harm and the resulting claims for damage compensation, or at least to minimize the likelihood of such an event. This is true only as long as such preventive measures are possible and efficient. In some cases, insurance solutions are chosen so as to allocate the risk and reduce the costs. Yet, the less predictable damages become, the more systemic effects are to be expected or the greater the scale of damages is that might occur, the more difficult is becomes to implement such insurance solutions. It represents another field in which legal regulation can be used to mitigate such effects or to prevent them entirely.

#### Temporary decommissioning

Service robots are generally used in a specific environment with a specific purpose, to aid a specific person or to perform a specific task. These circumstances usually come to an end at some point, either with the recovery or decease of a person in need of care, the liquidation of a business or simply the abandonment of the task that the service robot had been used for. The conditions under which the service relationship is disbanded can be special when it comes to the employment of service robots, considering that special customizations are demanded of the service robot which might prevent a termination on short notice. Both parties—on the part of provider and user—may have reasons to furnish the relationship with aftereffects and expiration obligations in order to facilitate the transfer to others or to provide for the rearrangement or deletion of existing data, say because of its personal nature. This includes the possible reversal of measures that had bee taken to provide a robot-friendly environment.

The termination of the specific service does not render the robot generally unusable, but does so only for that particular service. It can therefore be reemployed in a similar, or possibly even in a completely different context.

This reuse generates new issues that are subject to legal regulation. Generally speaking, the traceability of a robot is another problematic area for the law. This concerns both physical recycling as well as the knowledge accumulated in the service robot. Do ownership, copyright or privacy law warrant further measures right up to the act of deletion (which entails a forfeiture of knowledge)? It is part of the altogether unresolved issue of whom knowledge and information can firmly be attributed to, whether it can be owned at all and if so, who the owner is. Say a service robot is employed in the area of nursing and its service is paid for by public health care, the question then arises: Who has the right to exploit the information stored in the robot? To what extent can this knowledge be transferred to another robot and possibly used for other purposes?

#### Final decommissioning/disposal

A robot’s normal operation usually ends with its general decommissioning, the disposal. In contrast to a change of user or a change of service area, where its capability of service remains unimpaired, the operational capability ends with its disposal. The process of reducing it to waste is subject to regulation as well. The decision about it also represents a decision about how the stored knowledge is to be assigned and put to use and what importance is ascribed to further development. Hence, the issue is also one of setting standards for the evaluation of the state of the art and determining when it is to be regarded as essentially outdated. Thus, the decision about the disposal as well as the decision maker becomes crucial: Who has the final call concerning the utilization of the service robot, for instance in a healthcare system based on medical insurance? What are the standards and conditions for the decommissioning?

Through decommissioning, the service provider turns into an item that needs to be disposed of. Therefore, the legal provisions governing waste become applicable and we have come full circle to the developmental requirements: Waste legislation can and ideally should stipulate which materials, substances, and processes can be used for development in the first place. Likewise, the obligations of the last user and last provider materialize at this point: What particularities in the decommissioning process need to be observed, e.g. concerning material responsibilities, data storage and deletion or return?

### The adaption of prevailing law to the particularities of service robots using the example of autonomy

It has become apparent that the particularities of service robots are not sufficiently pronounced to make the development of an independent robot law worthwhile. Just the same, there are a row of issues that keep coming up at all stages of a systemic analysis of service robotics. The aspect of practical and legal autonomy seems to be of special significance across the board. It combines technical (what can a robot “decide on its own”?), philosophical (when does a robot “act autonomously”?), and psychological questions (when does the user perceive a robot to be acting “autonomously”?).

From a legal perspective, one ought to begin by differentiating between a “natural person” and a “legal entity.” The reasoning behind the establishment of legal entities lies in their ability to limit liability for economically desirable accumulations of capital; the legal entity’s liability and capacity to act is ensured by the imputation of so-called organ conduct, meaning that it is traced back to human behavior. This serves to illustrate that a constructed legal personality is not alien to law. This line of thought could be followed—especially in the case of systemically operating service robots such as advanced driver assistance systems—to the point of providing a service robot, it being an “electric person,” with a similar construction.

Without a legal notion of the division of labor, responsibility, and organization, the construction of imputing alien conduct to a person would not be possible. The law therefore provides for liability and supervisory obligations in respect to third party negligence, for example, parents are liable for the conduct of children, the principal is liable for the conduct of employees and subcontractors (agents/vicarious agents; special provisions in labor law). The imputation when using technical tools, instruments, and aids is much more straightforward. Their employment is routinely imputed to their user; in addition, manufacturers and operators are sometimes held liable as well.

The question of imputation is particularly pertinent when using robots that are connected with each other through a system. The advanced driver assistance system, for example, can make decisions that cannot be ascribed to a single vehicle, leading to considerable consequences for the individual participants. This is true especially when the navigated means of transportation are not at the center of attention, but rather just forms part of a general public driving system. Concepts of comparative negligence can only induce successful steering effects when the risks that might materialize can be covered by the typically recoverable assets of individuals. Otherwise, an insurance solution must shoulder the risk of insolvency.

These problems tie in with a range of already led discussions, for example, involving the legal aspects of adaptive software tools such as software agents or concerning complex systems of electromobility containing a multitude of autonomously operating components. Also, in the case of automated platforms with the help of which multiple actors—without entering into any kind of contractual relationship with each other—make information available that has been aggregated by a third party, the aggregated data can no longer be ascribed to specific actors. These approaches can be taken up and elaborated upon for service robots. Provided that service robots are capable of learning and interacting with their surroundings in ways that elude detailed predictability, a solution is conceivable where the conduct of specific robots is no longer attributed to their operator/user—if necessary by means of comparative fault—, but a distinct legal “accountability” of these novel machine “creatures” is assumed instead. The problem that would still remain is that the final result that has been “negotiated” by multiple agents can never be ascribed to a single agent, but always to several, yet never to any of these in its entirety.

This issue is paramount and urgent when it comes to liability for error and the distribution of liability risk. Hence, philosophical and ethical questions concerning the “autonomy” of agents only play a marginal role at best. The picture is quite different when it comes to dealing with questions such as: To what extent should law follow ethical and/or philosophical definitions and distinctions and include them in the body of law, or even base the body of law upon them? Is the law bound to its own normative standards, should it even be?

Closely related to the issue of autonomy and its legal framework is the issue of regulating the exercise of individual liberty from the decision to activate right up to the individual decommissioning. To what extent does the individual decision to activate/integrate a robot remain with the individual? In what way can interests of general welfare justify a paternalistic approach?

## Conclusion and outlook

A uniform body of law for service robotics does not appear practical at the present time. In light of the vastly different possible areas of employment for service robots and the diverse pool of services that robots could perform and assist with, an all-encompassing regulation would be quite unsuitable. Generalizations would be necessary, yet would scarcely lead to satisfactory result at this stage. Instead, provisions specific to service robots should be introduced into the respective areas of law wherever they are deemed appropriate, for example, in public health law and social (welfare) law, in construction and traffic law, in construction planning law or in the German Civil Code.

Law itself requires that an eye be kept on the further development of service robots. The monitoring and regulated examination of the legal and factual circumstances must be legally secured.

Liability rules have proven—particularly under uncertain conditions—a serviceable tool in enabling the development of potentially dangerous technology while at the same time having the profiteer shoulder part of the negative effects that might ensue. A liability system of this nature for the use of service robots represents an important instrument of control, especially considering the difficulties of attribution in partly autonomous systems. Such a liability system should not—similarly to genetic technology and nuclear law—be based solely on strict liability, but should rather incorporate a principle of fault. Otherwise, one might face the danger of excessive regulation that would compromise development in this field, especially in the private sector. One could, however, consider the introduction of mandatory liability insurance so as to absorb the damages sustained by third parties. In order to be able to establish standards for negligence, certain basic safety rules must be in place in private and freely accessible public space which the operators of service robots have to comply with. The problem of formulating such standards does not differ from standard-setting in other areas with highly dynamic development.

The connection of service robots to a (partly) autonomous system may well be desirable from an efficiency standpoint. However, every network leads to the problems of monopolization and dependency. The same applies to the interface between multiple systems. The formation of systems must therefore be accompanied by regulatory action, as is already the case in other areas of economic regulation. Hence, technology law must invariably be conceived of as economic law.
